# Coral‐Biomimetic Structured Organic Electro‐Oxidations With Exceptional High‐Current Catalytic Performance

**DOI:** 10.1002/advs.78182

**Published:** 2026-10-08

**Authors:** Chaoqun Pei, Tao Zhang, Congrui Yang, Yutao Tan, Zhengjie Chen, Yuyang Qian, Xiaoyu Wu, Xiaodi Liu, Xiong Liang, Jie Dong, Yongbin Guo, Haibo Ke, Wenqing Ruan, Zhenxuan Zhang, Tao Feng, Jing Peng, Jiang Ma, Baoan Sun

**Affiliations:** ^1^ Shenzhen Key Laboratory of High Performance Nontraditional Manufacturing College of Mechatronics and Control Engineering Shenzhen University Shenzhen People's Republic of China; ^2^ Institute of Physics Chinese Academy of Sciences Beijing People's Republic of China; ^3^ Songshan Lake Materials Laboratory Dongguan Guangdong People's Republic of China; ^4^ Faculty of Materials Science and Energy Engineering/Institute of Technology for Carbon Neutrality Shenzhen Institute of Advanced Technology Chinese Academy of Sciences Shenzhen People's Republic of China; ^5^ Institute of Automation Qilu University of Technology (Shandong Academy of Sciences) Jinan Shandong People's Republic of China; ^6^ Dongguan Institute of Materials Science and Technology Chinese Academy of Sciences Dongguan Guangdong People's Republic of China; ^7^ Herbert Gleiter Institute of Nanoscience School of Material Science and Engineering Nanjing University of Science and Technology Nanjing People's Republic of China

**Keywords:** amorphous/crystalline heterostructures, biomimetic structure, hydroxymethylfurfural oxidation reaction, organic electrooxidation reaction, urea oxidation reaction

## Abstract

Organic electrooxidation reactions (OEORs) provide a thermodynamically favorable pathway for hydrogen production by replacing the oxygen evolution reaction with more readily oxidized organic substrates. However, the development of OEOR catalysts that simultaneously achieve high activity, stability, and energy efficiency at high current density remains a fundamental challenge. Herein, we report a bio‐inspired multiscale catalyst (Cu_x_O@CuNiFeMoP/3D‐S) with a coral‐like hierarchical architecture, fabricated via integration of 3D‐printed triply periodic minimal surface scaffolds and pulse‐electrodeposited amorphous/crystalline heterostructures. This catalyst exhibits record‐breaking performance in the hydroxymethylfurfural oxidation reaction (HMFOR), requiring low potentials of 1.47 V at 500mA cm^−2^ and 1.57 V at 1000 mA cm^−2^ while maintaining 99.5% conversion. We identify a dual‐copper synergistic mechanism, where the coexistence of Cu^+^/Cu^2+^ together with Ni sites enables efficient catalysis under high current density. The mechanism demonstrates universality in diverse OEORs, especially achieving 500 mA cm^−2^ at 1.34 V for the urea oxidation reaction. By coupling OEORs with hydrogen evolution, this work provides an integrated architectural and mechanistic framework for designing multifunctional catalysts, enabling energy‐efficient hydrogen production with concurrent biomass valorization.

## Graphical abstract

A strategy for energy‐efficient hydrogen production coupled with biomass valorization via organic electrooxidation reactions is reported. A bio‐inspired multiscale coral‐like heterostructure catalyst delivers record‐breaking OEOR performance enabled by a dual‐copper synergistic Cu^+^/Cu^2+^–Ni mechanism, establishing a universal architectural and mechanistic framework for multifunctional high‐current‐density electrocatalysis.

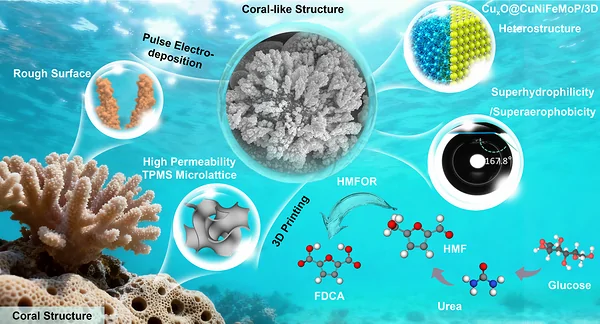

## 1 Introduction

Hydrogen (H_2_) has risen to the forefront of renewable energy carriers amid the global push toward carbon neutrality, with water splitting identified as the critical technology underpinning its sustainable production [1, 2]. However, conventional water splitting, relying on the anodic oxygen evolution reaction (OER) and cathodic hydrogen evolution reaction (HER), faces inherent bottlenecks that undermine its scalability and cost‐effectiveness [3, 4]. Thermodynamically, water splitting needs a minimum potential of 1.23 V, yet practical commercial systems operate at much higher voltages (1.6 – 2.0 V). This discrepancy arises from the large overpotential of OER, which accounts for over 80% of total electricity consumption and sluggish reaction kinetics [5, 6, 7]. In addition, the slow evolution kinetics of H_2_/O_2_ gases lagging behind their electrolyte membrane permeation causes cross‐mixed gases and explosive mixture risk [8], while the low‐value OER product (O_2_) and O_2–_catalyst‐derived reactive oxygen species that degrade membranes further raise maintenance costs and sudden production shutdown risks [9]. To circumvent these limitations of conventional water electrolysis, the replacement of OER with organic electrooxidation reactions (OEORs) (e.g., 5‐hydroxymethylfurfural oxidation reaction (HMFOR) [10], urea oxidation reaction (UOR) [11], glucose oxidation reaction (GOR) [12], et al.) at the anode has emerged as a transformative strategy. This approach offers two critical advantages: First, OEORs exhibit substantially lower theoretical potentials than those of conventional OER (e.g., HMFOR ∼0.6 V, UOR ∼ 0.37 V, GOR ∼ 0.05 V) [13, 14, 15, 16], directly reducing the overall cell voltage and energy consumption for H_2_ production. Second, the absence of O_2_ in the OEOR not only avoids the explosion risk associated with the mixing of H_2_, CH_4_, and CO with O_2_, but also enables the production of high‐value‐added chemicals at the anode, thereby boosting economic benefits [17].

Despite these merits, OEOR‐assisted H_2_ production remains constrained by fundamental challenges that impede industrial adoption. A pivotal bottleneck for industrial application is inadequate electrocatalytic performance at high current density. Most reported OEORs, for instance, only exhibit viable activity under low current densities (< 100 mA cm^−2^), a value far below the threshold (> 500 mA cm^−2^) required for industrial application [18, 19, 20, 21]. Their inability to enable rapid mass transfer and product desorption at high current densities further leads to active site coverage by products. For instance, nickel‐based catalysts are widely investigated for OEORs [22]. However, even modified nickel‐based systems, such as Fe‐doped Ni‐Fe layered double hydroxides, which enhance proton transfer via lattice distortion, still struggle to improve high‐current‐density performance [23]. They exhibit excellent activity at low current densities but suffer from excessively high overpotentials under high‐current conditions. Similarly, Cu‐based catalysts display low overpotentials at low currents but fail to retain activity at high currents due to the unstable Cu^3+^ active center originating from the intermediates‐blocked Cu active site, rendering them incompatible with the demands of industrial‐scale H_2_ production [24, 25, 26]. Furthermore, the complex multi‐electron‐transfer processes for typical OEORs, for example HMFOR to FDCA (6e^−^), complete UOR process (6e^−^), and GOR (2e^−^ for gluconic acid, 6e^−^ for glucaric acid, up to 24e^−^ for full mineralization to CO_2_) [27, 28, 29, 30], create inherent kinetic barriers, increasing the reaction overpotential and further exacerbating the difficulty of clarifying reaction mechanisms and optimizing catalyst performance. In addition, the high‐concentration organic solutions (>50 mm) necessary for industrial processes such as HMFOR electrolysis substantially increase viscosity, thus impairing fluidity, hindering mass transfer, and accelerating competing side reactions [31, 32, 33]. Therefore, developing an electrocatalyst that delivers high activity under industrially relevant high‐current‐density conditions is crucial to advancing OEORs toward practical hydrogen production applications.

To address the persistent challenges, we develop a multi‐scale biomimetic catalyst (Cu_x_O@CuNiFeMoP/3D‐S) with coral‐like architecture that maintains exceptional activity at high current density across universal OEORs. Inspired by natural coral systems—which integrate porous reef substrates with micro‐rough branches—we construct an artificial catalytic system through synergistic combination of 3D‐printed triply periodic minimal surface (TPMS) scaffolds and pulse‐electrodeposited coral‐like branches serving as highly accessible active sites. The resulting architecture features an amorphous CuNiFeMoP matrix embedded with face‐centered cubic (FCC) Cu_2+1_O (Cu_x_O) nanocrystals, forming a highly active heterostructure. In HMFOR, the Cu_x_O@CuNiFeMoP/3D‐S catalyst achieves breakthrough performance, requiring only 1.42 to reach 10 mA cm^−2^ (1.63 V for commercial RuO_2_) and merely 1.57 V to achieve an industrial‐level current density of 1000 mA cm^−2^. Simultaneously, it enables near‐quantitative HMF conversion of 99.5%, highlighting its practical potential. Mechanistic investigation reveals a novel dual‐copper synergy mechanism, where the coexistence of Cu^+^/Cu^2^^+^ species collaborates synergistically with Ni sites within the heterostructure, facilitating enhanced catalysis under high‐current conditions. Remarkably, this mechanism demonstrates universal applicability in diverse OEORs (GOR and UOR), as evidenced by exceptional UOR performance achieving 500 mA cm^−2^ at only 1.34 V—positioning it among the top‐performing UOR electrocatalysts reported to date. This work establishes a versatile catalytic platform that transcends substrate‐specific limitations and provides an efficient strategy for industrial organic‐assisted electrolytic hydrogen production.

## 2 Results and Discussion

The multi‐scale architecture of marine coral ecosystems offers profound biomimetic insights for designing advanced electrocatalysts. In natural coral systems, a synergistic combination of porous reef substrates and textured branches forms an exceptionally efficient platform for mass transport and energy conversion (Figure 1a). The three‐dimensionally interconnected channel networks within coral substrates not only provide mechanical support for coral growth but also facilitate rapid transport of marine nutrients and metabolic products through low‐resistance pathways. Concurrently, the surface morphology of the coral‐branch structure, as characterized by scanning electron microscopy (SEM), demonstrates a highly rough texture with prominent protrusions, significantly enhancing the specific surface area (Figure S1). The micro‐nano structured surfaces of coral branches enhance interfacial processes through significantly increased specific surface area that collectively optimize mass exchange efficiency at solid‐liquid interfaces [34]. This inherent “macro‐transport/micro‐reaction” synergy presents striking parallels with critical processes in electrocatalysis, including reactant diffusion, interfacial reactions, and product desorption.

**FIGURE 1:**
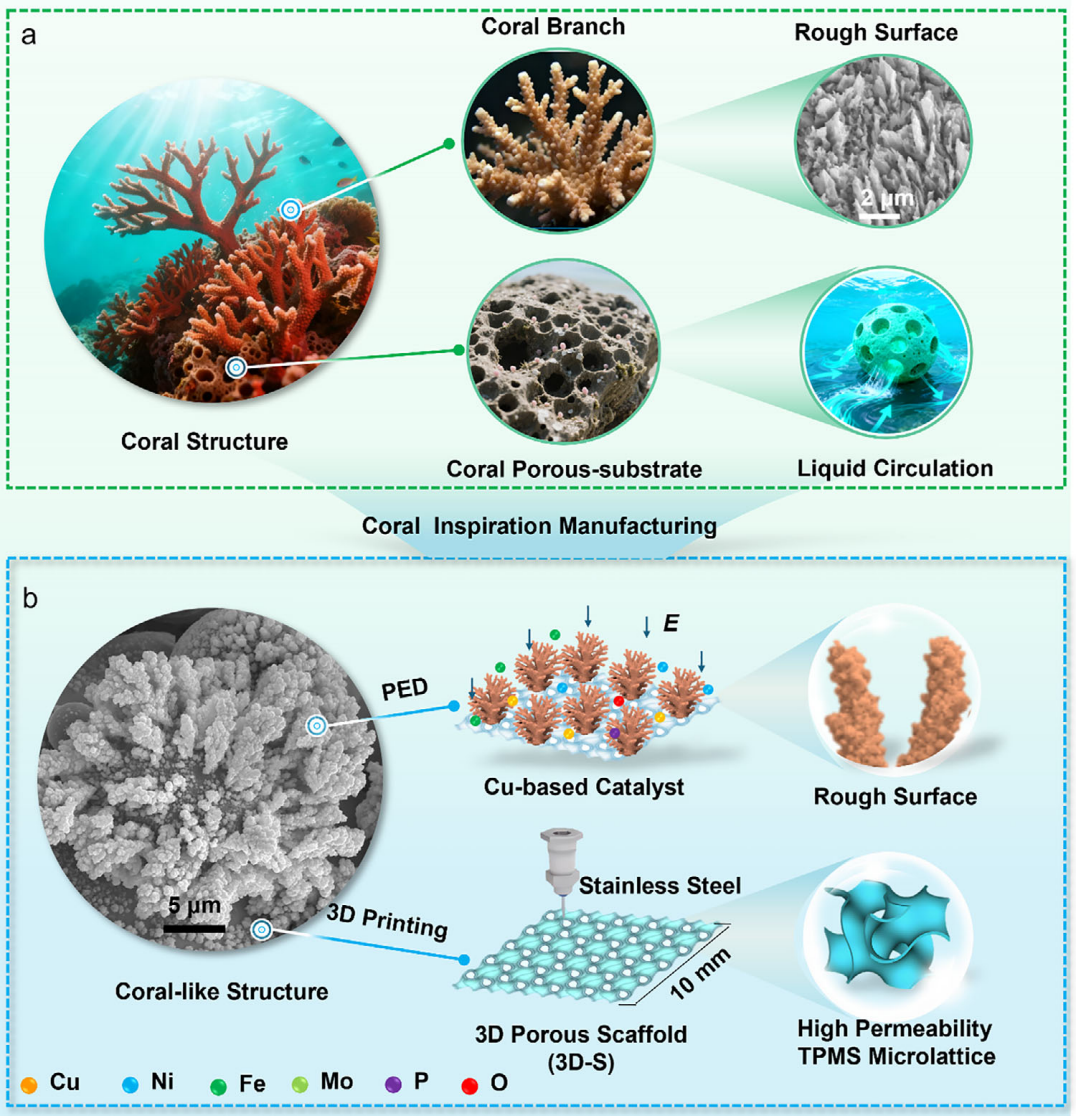
Schematic diagram of the manufacture of monolithic Cu_x_O@CuNiFeMoP/3D‐S hierarchical catalyst with coral‐inspired biomimetic structures via 3D printing and PED. (a) The structure of the natural coral system. (b) Design of monolithic Cu_x_O@CuNiFeMoP/3D‐S hierarchical catalyst with coral‐inspired biomimetic structure.

Building upon these structure‐function correlations, we have innovatively integrated coral‐inspired hierarchical design with metamaterial engineering principles to develop a new class of biomimetic electrocatalytic materials (Figure 1b). Our methodology involves two key bio‐inspired strategies: First, emulating the porous architecture of coral reefs that combines robustness with efficient matter transfer, we employ 3D‐printed stainless‐steel TPMS scaffolds (10 mm × 10 mm × 0.5 mm) that offer a scalable, reproducible structural framework (Figures S2 and S3a). TPMS structures, characterized by continuous and highly interconnected pore networks, provide efficient three‐dimensional pathways for electrolyte transport. Flow‐field simulations reveal that the electrolyte is broadly distributed throughout the interconnected channels (Figure S4). Further analysis of the local porosity and the velocity components along the *x*, *y*, and *z* directions shows that the spatially varying pore geometry induces repeated flow acceleration/deceleration and pronounced three‐dimensional flow redirection, thereby promoting convective electrolyte redistribution and continuous renewal of the liquid phase adjacent to the catalytic surfaces (Figure S5). Meanwhile, their geometry‐dependent stiffness provides excellent mechanical robustness (Figure S6). These combined advantages make TPMS structures ideal load‐bearing and mass‐permeable backbones for high‐performance electrocatalysts (Figure 1b).

Second, mimicking the surface characteristics of coral branches, we utilize pulsed electrodeposition (PED) to co‐deposit multiple metallic elements (Cu, Fe, Ni, Mo, P) onto TPMS scaffolds (3D‐S), creating active interfaces with coral‐like micro‐nano roughness (Figure 1b and Figure S3b). These biomimetic surfaces not only dramatically increase the density of accessible active sites but also establish a favorable solid–liquid–gas interfacial environment. We further investigated the hydrophilicity and aerophobicity properties of the coral‐like Cu_x_O@CuNiFeMoP/3D‐S catalyst, as summarized in Figure S7. Contact angle measurements (Figure S7a,b) reveal a dramatic transformation from the inherently hydrophobic 3D‐S substrate (water contact angle 126.7°) to a nearly perfect super‐hydrophilic state following catalyst deposition. The modified surface exhibits a near‐zero water contact angle with complete droplet absorption occurring within 6 ms, indicating instantaneous wetting behavior crucial for rapid electrolyte access to active sites. Complementary bubble contact angle characterization demonstrates equally remarkable aerophobic characteristics (Figure S7c,d). While the bare 3D‐S substrate shows moderate bubble adhesion (151.9°), the Cu_x_O@CuNiFeMoP/3D‐S architecture achieves superior bubble repellency with a contact angle of 167.8°—approaching the theoretical limit for non‐sticking behavior. These interfacial characteristics favor rapid electrolyte infiltration and gas‐bubble removal, thereby maintaining effective reactant supply and product release during electrocatalysis. This cross‐scale biomimetic design, achieved through the synergistic integration of 3D printing and PED, enables precise structural control spanning from macroporous transport networks to micro–nano surface features. The resulting coral‐like hierarchical catalysts therefore combine architecture‐regulated convective transport, rapid electrolyte infiltration, favorable gas removal, and abundant active sites with optimized interfacial environments.

Comprehensive structural characterization of the synthesized hierarchical Cu_x_O@CuNiFeMoP/3D‐S coral‐like catalyst is conducted in Figure 2. Figure 2a displays a macroscopic view of the catalyst specimen, presenting a 10 mm × 10 mm sheet structure. The image reveals two distinct regions: Region I (lighter area) corresponds to the 3D‐S, while Region II (darker area) represents the coral‐branch architecture of Cu_x_O@CuNiFeMoP deposited via PED. Higher‐magnification SEM image (Figure 2b) reveals that Region II exhibits micrometer‐scale porous networks and curved surfaces originating from the deliberately designed TPMS architecture of the 3D‐S inspired by a coral porous substrate. The PED‐derived coral‐branch structures uniformly coat the 3D‐S surface, forming a continuous hierarchical framework. Further magnification (Figure 2c,d) reveals that the coral branches possess remarkably rough surfaces with nanoscale texture. Elemental analysis via EDS confirms that the coral branches consist of Cu, Ni, Fe, Mo, and P elements in a multi‐metallic composition. Transmission electron microscopy (TEM) investigations (Figure 2f,g) unveil an intriguing nanoscale dual‐phase heterostructure within the branches. High‐resolution TEM (HR‐TEM) (Figure 2g) distinguishes two distinct regions: blue areas corresponding to amorphous CuNiFeMoP domains and yellow regions comprising highly crystalline nanoparticles. Combining HR‐TEM and fast Fourier transform (FFT) analysis of the nanocrystalline regions (in Figure 2h,i) identifies them as FCC‐structured Cu_2+1_O crystals along the [0, ‐1, ‐1] zone axis. The measured interplanar spacing (*d*) of 2.09 Å corresponds to the (200) planes of Cu_2+1_O [35, 36]. Further x‐ray diffraction (XRD) characterization of the catalyst (Figure S8) confirms the presence of Cu_2+1_O, consistent with TEM analysis. However, the strong diffraction signal from the 3D‐S scaffold dominates the pattern and obscures the characteristic broad halo of the amorphous matrix. Complementary XPS analysis of Cu (Figure 2j) reveals three distinct states: Cu^2+^ (binding energy (BE) of 934.1 eV) and Cu^+^/ Cu^0^ (932.8 eV), indicating the coexistence of mixed‐valence copper species [37]. To further verify the coexistence of Cu^+^ and Cu^2+^ identified by XPS, Cu K‐edge XANES measurements of Cu_x_O@CuNiFeMoP were performed (Figure S9). The absorption edge of Cu_x_O@CuNiFeMoP lies between those of CuO and Cu_2_O, confirming the mixed Cu^+^/Cu^2+^ nature, which matches our XPS analysis. This finding aligns perfectly with the TEM observations of dual‐phase nanostructures. Collectively, these results demonstrate the successful fabrication of a multi‐level composite catalyst through the synergistic combination of 3D printing and PED technologies. The electrodeposited copper‐based coral branches exhibit sophisticated nanoscale dual‐phase heterostructures, featuring amorphous matrices embedded with crystalline Cu_2+1_O nanoparticles, while maintaining mixed copper valence states that are potentially crucial for catalytic functionality.

**FIGURE 2:**
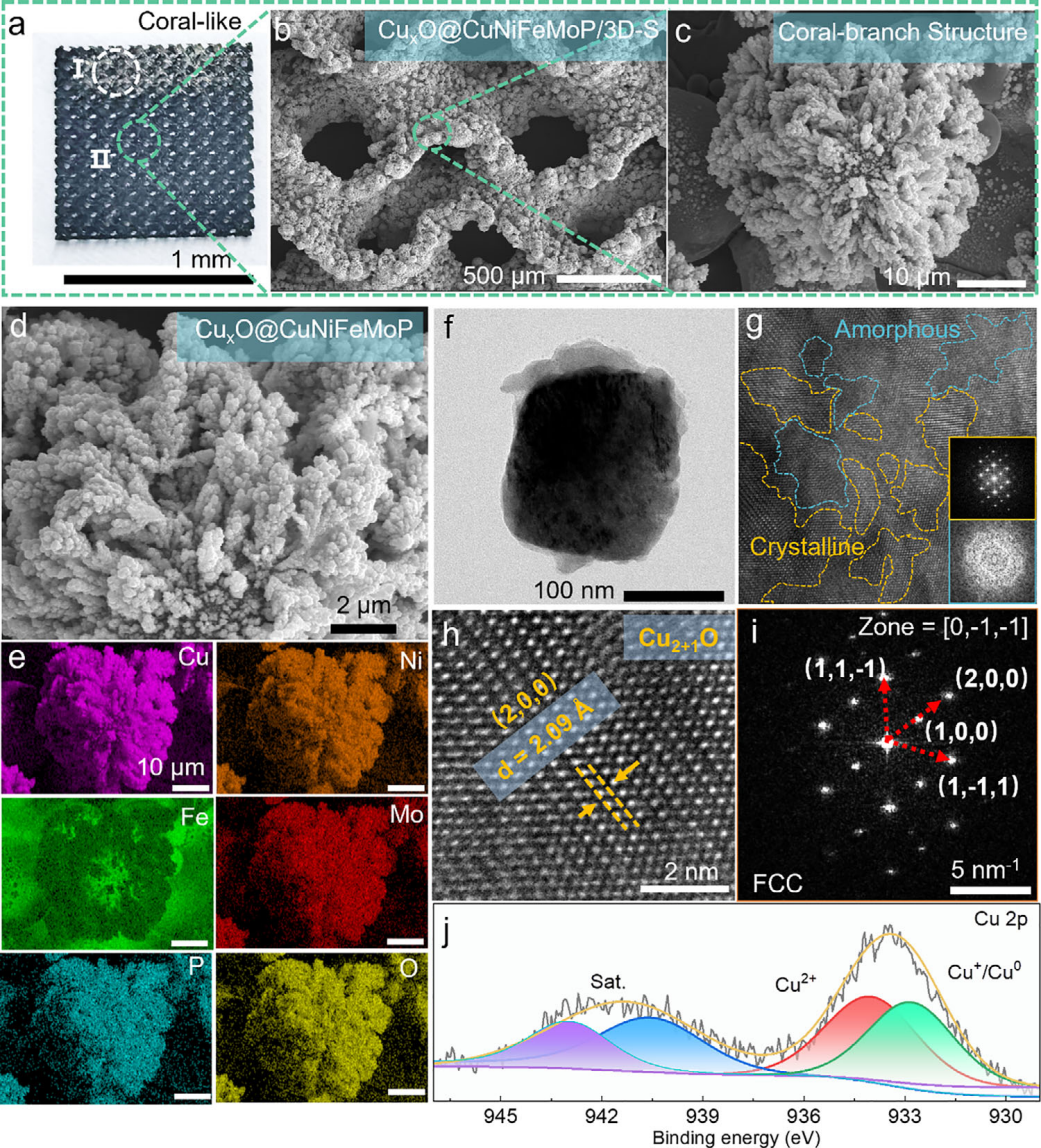
Structural characterizations of the hierarchical Cu_x_O@CuNiFeMoP/3D‐S coral‐like structured catalyst. (a) Image of the Cu_x_O@CuNiFeMoP/3D‐S coral‐like structured catalyst. (b–d) SEM images of Cu_x_O@CuNiFeMoP/3D‐S with gradual magnification of region II from (a). (e) Corresponding EDS results of Cu_x_O@CuNiFeMoP from (d). (f) TEM observation area of Cu_x_O@CuNiFeMoP. (g) HRTEM image of Cu_x_O@CuNiFeMoP. (h) High‐resolution result of the crystalline region from (g). (i) FFT result corresponding to the Cu_2+1_O nanocrystalline from (h). (j) High‐resolution XPS spectrum of Cu of Cu_x_O@CuNiFeMoP/3D‐S catalyst.

The OEOR performance of the hierarchical Cu_x_O@CuNiFeMoP/3D‐S catalyst was systematically evaluated for the HMFOR, as summarized in Figure 3. Figure 3a illustrates the proposed HMFOR pathways, highlighting two primary routes for HMF conversion to the intermediate 5‐formyl‐2‐furancarboxylic acid (FFCA): pathway (1) proceeds through 5‐hydroxymethyl‐2‐furan carboxylic acid (HMFCA) formation, while pathway (2) involves 2,5‐furan dicar‐boxaldehyde (DFF) as the key intermediate, with both routes ultimately yielding 2,5‐furandicarboxylic acid (FDCA) as the final product [38]. Linear sweep voltammetry (LSV) in 1 m KOH + 50 mm HMF (Figure 3b) demonstrates that Cu_x_O@CuNiFeMoP/3D‐S requires only 1.42 V to achieve 100 mA cm^−2^, significantly lower than commercial RuO_2_ (1.63 V), conventional Ni(OH)_2_ (1.47 V, Figure S10), and the bare 3D‐S scaffold. More importantly, the catalyst maintains outstanding activity under industrially relevant conditions, reaching 500 and 1000 mA cm^−2^ at remarkably low potentials of 1.47 and 1.57 V, respectively (Figure 3c). Furthermore, Cu_x_O@CuNiFeMoP/3D‐S exhibits superior catalytic performance compared with the conventional Ni foam skeleton (Cu_x_O@CuNiFeMoP/NF), especially at high current densities (Figure S11), further confirming the beneficial effect of 3D‐S on boosting catalytic performance. To clarify the necessity of introducing multiple metallic components, CuNiMoP and CuNiP catalysts were also fabricated on the same 3D‐S. The corresponding LSV curves are presented in Figure S12. At a high current density of 500 mA cm^−2^, the CuNiFeMoP, CuNiMoP and CuNiP catalysts show potentials of 1.47, 1.64 and 1.67 V, respectively. These results verify that the quinary CuNiFeMoP catalyst exhibits distinctly superior HMFOR activity compared with ternary CuNiP and quaternary CuNiMoP counterparts. Kinetic analysis through Tafel plots (Figure 3d) further confirms superior performance, with Cu_x_O@CuNiFeMoP/3D‐S exhibiting a slope of 42.15 mV dec^−1^, substantially lower than RuO_2_ (98.36 mV dec^−1^) and 3D‐S (79.24 mV dec^−1^), indicating enhanced reaction kinetics. The intrinsic activity of the catalysts is also compared after ECSA normalization (Figure S13). Comparative analysis with recently reported HMFOR catalysts (Figure 3e) positions Cu_x_O@CuNiFeMoP/3D‐S among the most active systems, particularly notable for its low‐voltage operation in the industrial current‐density regime. Product distribution analysis during electrolysis (Figure 3f,g) reveals near‐ complete HMF consumption with simultaneous FDCA formation approaching the theoretical maximum yield. The consistently minimal DFF concentration throughout the reaction suggests preferential progression through pathway 1 (Figure 3a), consistent with the reported reaction route under alkaline conditions. Furthermore, delivery of ∼144.7 C (near‐quantitative HMF oxidation efficiency under the prevailing kinetic competition) eradicates the HMF signature while maximizing the FDCA response, signifying quantitative conversion (Figure 3f,g and Figures S14 and S15). Open circuit potential (OCP) is monitored to semi‐quantitatively probe the HMF population within the inner Helmholtz plane (IHP) [39]. Upon injection of 50 mm HMF, all catalysts exhibited an instantaneous OCP drop; the magnitude of the negative shift for Cu_x_O@CuNiFeMoP/3D (∆*E =* 0.50 V) significantly exceeded that of undoped Substrate (∆*E =* 0.05 V), evidencing the enhanced HMF surface coverage in the IHP (Figure 3h). Most remarkably, as shown in Figure 3i, the catalyst achieves 95.7% HMF conversion at low current density (1.42 V @ 100 mA cm^−2^), and this efficiency not only maintains but improves to 99.5% under high‐current‐density operation (1.47 V @ 500 mA cm^−2^). Notably, the selectivity toward FDCA ranks among the highest values reported for HMFOR electrocatalysts to date (in Figure S16). Although a decline in FDCA Faradaic efficiency is observed at higher current densities (1.57 V @ 1000 mA cm^−2^) owing to the activation of the competing OER (Figure S17), this does not fundamentally compromise the hydrogen‐production voltage‐reduction effect. This represents a significant advancement toward practical implementation of organic‐assisted water electrolysis systems.

**FIGURE 3:**
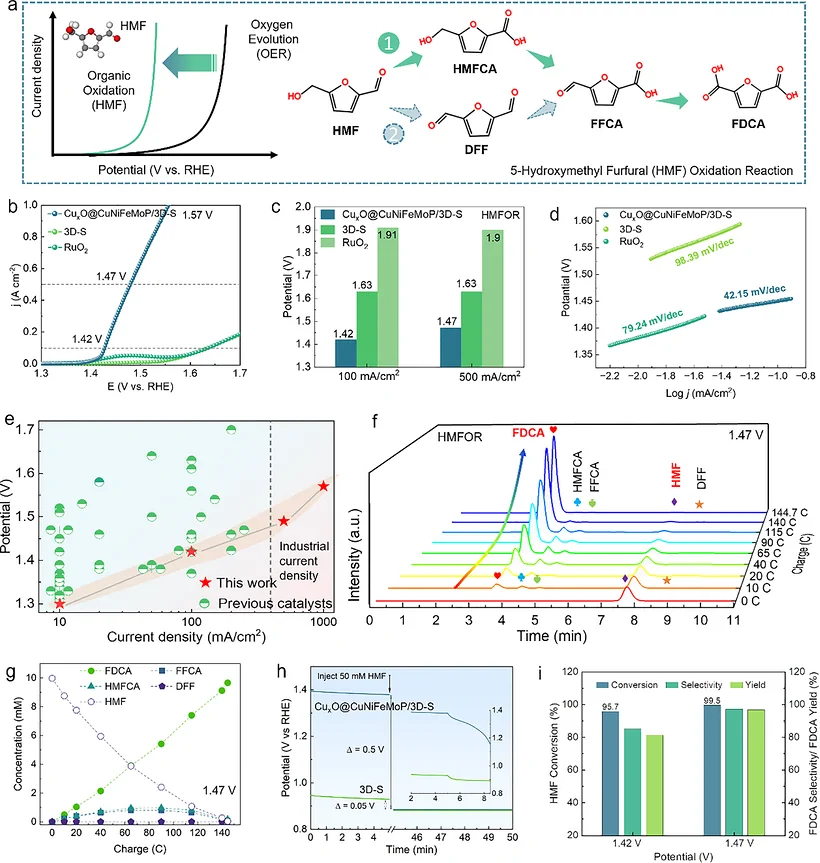
HMFOR Performance of the hierarchical Cu_x_O@CuNiFeMoP/3D‐S catalyst. (a) Schematic diagram of the reaction pathways of the HMFOR. (b) LSV curves of Cu_x_O@CuNiFeMoP/3D‐S, 3D‐S, and RuO_2_ catalysts for HMFOR. (c) Histogram of potentials at 100 and 500 mA cm^−2^ for different catalysts for HMFOR. (d) Tafel curves of Cu_x_O@CuNiFeMoP/3D‐S, 3D‐S, and RuO_2_ catalysts for HMFOR. (e) Comparison of potentials at different current densities of the Cu_x_O@CuNiFeMoP/3D‐S catalyst for HMFOR. (f) Time‐resolved chromatograms tracked the temporal evolution of these species throughout electrolysis at the potential of 1.47 V. (g) Concentration of different reaction products of Cu_x_O@CuNiFeMoP/3D‐S during HMFOR at a potential of 1.47 V. (h) OCP curves of Cu_x_O@CuNiFeMoP/3D‐S and 3D‐S for HMFOR. (i) HMF conversion and FDCA selectivity/FDCA yield of Cu_x_O@CuNiFeMoP/3D‐S at different potentials.

To unravel the origin of the synergistic catalytic behavior in HMFOR of Cu_x_O@CuNiFeMoP/3D‐S across low and high current densities, we conducted comprehensive mechanistic characterization through coupled in situ spectroscopic and electrochemical analyses. Figure 4a,b present the in situ electrochemical impedance spectroscopy (EIS) results of Cu_x_O@CuNiFeMoP/3D‐S during OER and HMFOR under different applied potentials. In the in situ OER tests (Figure 4a), we observe a distinct peak in the phase angle (white dashed arrow) at potentials between 1.00 and 1.30 V, corresponding to the catalyst oxidation process. As the potential increases to 1.60 V, a peak emerges in the low‐frequency region (0.1–1 Hz) and gradually diminishes, representing the OER process. In contrast, the EIS results for HMFOR (Figure 4b) show significant differences. While the same catalyst oxidation peak (white dashed arrow) appears between 1.00 and 1.30 V, an additional anomalous oxidation peak emerges at 1.40 V – 1.45 V (0.01–1 Hz), indicating the occurrence of HMFOR—consistent with the results in Figure 3b. Thus, in situ EIS measurements provide direct evidence that HMFOR occurs prior to OER. Furthermore, we performed in situ Raman spectroscopy during both OER and HMFOR processes (Figure 4c,d). For OER (Figure 4c), two peaks appear at approximately 290.6 and 337.2 cm^−1^ at low potentials, corresponding to Cu^2+^─O bonds. The peaks located at 521 cm^−1^ and 623.6 cm^−1^ can be attributed to the vibrations of Cu^+^─O bonds. This indicates the coexistence of Cu^2^^+^ and Cu^+^, which is consistent with the TEM results shown in Figure 2 [40, 41]. As the potential increases during OER, the copper‐related peaks disappear and Ni^3^^+^ oxidation peaks emerge, suggesting Ni^3^^+^ becomes the primary active site at high OER potentials [42, 43]. The in situ Raman results for HMFOR (Figure 4d) reveal that Cu^2+^ and Cu^+^ continue to coexist at low potentials, consistent with OER behavior. However, at high potentials (1.50 V – 1.60 V), both Cu^2+^ and Ni^3^^+^ oxidation peaks remain simultaneously present, indicating cooperative catalysis between Cu^2+^ and Ni^3^^+^ during HMFOR at high voltages, which explains the significantly enhanced catalytic activity [43]. Additionally, we conducted XPS analysis of HMFOR processes (Figure 4e,f). The binding energy of 932.8 eV is assigned to Cu^+^/Cu^0^ species, while the binding energy of 934.1 eV corresponds to Cu^2+^ (Figure 4e, OCP) [36, 37, 44]. At an applied potential of 1.4 V, the relative intensity of the Cu^+^ component increases, indicating partial oxidation of Cu^0^ to Cu^+^, consistent with Raman results. Upon further increasing the potential to 1.5 V, the relative peak intensity of Cu^2+^ rises remarkably, revealing the partial transformation from Cu^+^ to Cu^2+^. In general, higher applied potentials drive the oxidation of low‐valence Cu toward high‐valence Cu species, which is well corroborated by the in situ Raman results in Figure 4c,d. In addition, the Ni 2p XPS results (Figure 4f) demonstrate the existence of Ni^0^ under open‐circuit potential (OCP), which is derived from the amorphous domain of CuNiFeMoP. At high potentials, the fraction of Ni^3+^ increases substantially, which originates from the generation of catalytically active NiOOH species [45, 46]. This observation is also in good agreement with the in situ Raman spectra in Figure 4d.

**FIGURE 4:**
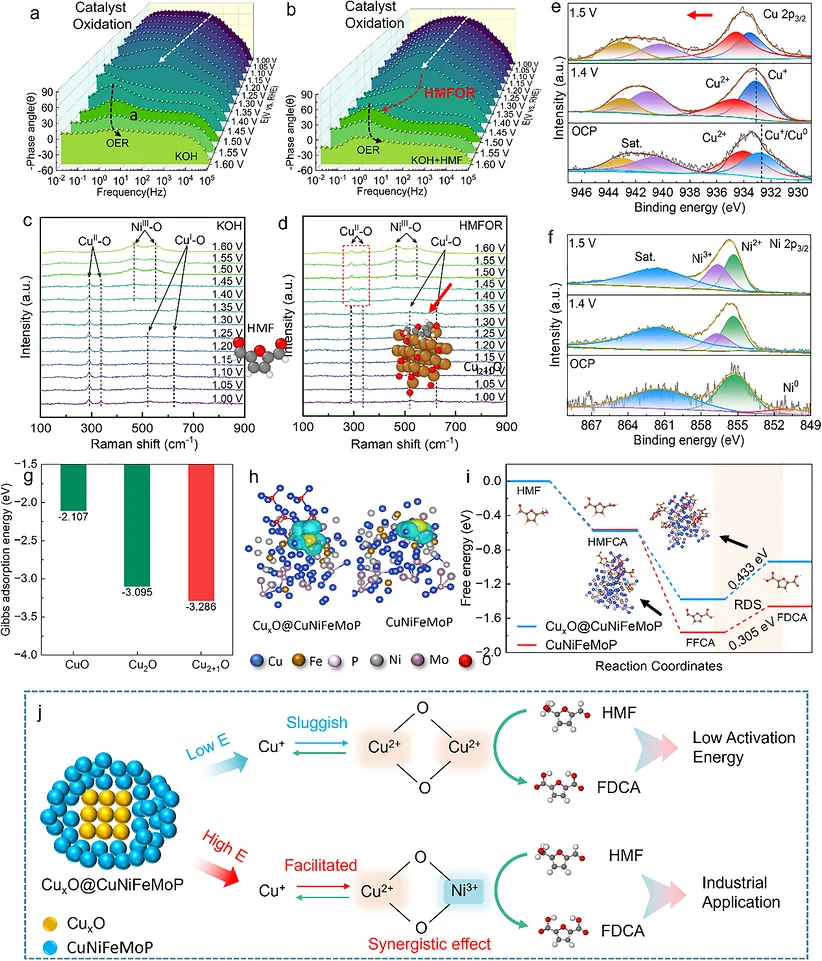
Comprehensive mechanistic characterizations of hierarchical CuxO@CuNiFeMoP/3D‐S catalyst for HMFOR. (a) In situ EIS results of Cu_x_O@CuNiFeMoP/3D‐S in OER. (b) In situ EIS results of Cu_x_O@CuNiFeMoP/3D‐S in HMFOR. (c) In situ Raman spectroscopy of Cu_x_O@CuNiFeMoP/3D‐S in OER. (d) In situ Raman spectroscopy of Cu_x_O@CuNiFeMoP/3D‐S in HMFOR. (e) XPS analysis of the Cu element of Cu_x_O@CuNiFeMoP/3D‐S in HMFOR. (f) XPS analysis of the Ni element of Cu_x_O@CuNiFeMoP/3D‐S in HMFOR. (g) Adsorption energies of Cu_2_O, CuO, and Cu_2+1_O for HMF. (h) Charge difference density of Cu_x_O@CuNiFeMoP and CuNiFeMoP. The blue and yellow areas represent the decrease and increase in charge density. (i) The structural evolution of catalyzed HMF electrooxidation. (j) Schematic diagram of the synergistic catalytic mechanism of Cu_x_O@CuNiFeMoP/3D‐S in HMFOR.

To unravel the origin of the enhanced catalytic performance, we performed DFT theoretical simulations (Figure 4g–i). The DFT calculations reveal that Cu_2+1_O exhibits stronger adsorption capability toward HMF compared with Cu_2_O and CuO (Figure 4g). We further compared the adsorption behaviors of HMF and OH^−^ on Cu_2+1_O (Figure S18). The results confirm that Cu_2+1_O preferentially adsorbs HMF molecules, which favors the HMFOR competitive reaction over OER and supports our experimental observations. Nevertheless, we emphasize that the HMFOR activity originates not solely from Cu_2+1_O, but also from the contribution of Ni sites. In situ Raman measurements verify the persistent presence of Ni‐related signals under high‐potential conditions (Figure 4d), which evidences the participation of Ni during electrocatalysis. This conclusion is further supported by DFT calculations. Two models were constructed: fully amorphous CuNiFeMoP and amorphous‐crystal hetero‐structured Cu_x_O@CuNiFeMoP (Figure 4h). After introducing the Cu_2+1_O component, obvious modulation of the electronic structure of Ni is observed at the heterointerface. The Ni d‐band center shifts upward by approximately 0.301 eV after inducing the amorphous‐crystal heterostructure of Cu_x_O@CuNiFeMoP (Figure S19a). The interfacial charge polarization around Ni is significantly enhanced; the local integrated charge variation of Ni reaches ∼2.16 times that of the pure amorphous counterpart (Figure S19b). These results indicate that the defective oxide phase (Cu_2+1_O) can tune the Ni 3d electronic configuration via interfacial charge rearrangement, adjusting the binding strength between Ni sites and HMF as well as reaction intermediates, and thus benefiting the HMFOR process. Moreover, we calculated the HMFOR reaction pathway (Figure 4i). The amorphous‐crystal Cu_x_O@CuNiFeMoP heterostructure reduces the energy barrier of the rate‐determining step (RDS) by 0.128 eV, which accounts for the major enhancement of catalytic performance. Collectively, integrating in situ EIS, Raman, DFT, and XPS results, we establish a novel cooperative catalytic mechanism for Cu_x_O@CuNiFeMoP/3D‐S during HMFOR across low and high current densities, as illustrated in Figure 4j. We propose that at low current densities, the coexistence of Cu^2+^ and Cu^+^ enables reversible redox reactions, where Cu^2+^ serves as the primary active site with low activation energy, explaining the low onset potential for HMFOR. The slow oxidation rate at low current density maintains the Cu^+^/Cu^2+^ equilibrium, detectable across multiple techniques. At high current densities, accelerated Cu^+^ to Cu^2+^ oxidation provides abundant active sites, making Cu^+^ undetectable at high potentials due to rapid turnover. Ni^3+^ activates as a catalytic site, requiring higher activation energy than Cu^2+^ and thus only becoming active at elevated potentials. Concurrently, the electronic structure of Ni is obviously modulated at the heterointerface, significantly reducing the reaction energy barrier for HMFOR. We conclude that at high current densities, activated Cu^2+^ and Ni^3+^ sites function cooperatively to maintain exceptional catalytic performance. Thus, we have discovered this novel synergistic catalytic mechanism operating across low and high current densities in the Cu_x_O@CuNiFeMoP/3D‐S catalyst system.

We further engineered a two‐electrode electrolyser system (TEE) that couples the low‐potential HMFOR with the cathodic HER, enabling simultaneous hydrogen production and value‐added FDCA synthesis (Figure 5a). The system comprises a peristaltic pump, separated anolyte (100 mm HMF in 1 m KOH) and catholyte (1 m KOH) streams, and a two‐electrode electrolyser featuring a serpentine flow field designed to enhance organic transport (Figure 5b). The anode and cathode consist of Cu_x_O@CuNiFeMoP/3D‐S and Pt/C composites, respectively. Polarization curves measured in the two‐electrode configuration (Figure 5c) demonstrate that Cu_x_O@CuNiFeMoP/3D‐S significantly outperforms the bare 3D‐S substrate under two‐electrode operation. At a current density of 100 mA cm^−2^, the Cu_x_O@CuNiFeMoP/3D‐S electrode requires a cell voltage of only 1.50 V, 400 mV lower than that of 3D‐S (1.90 V). More notably, under industrial‐level current density (1000 mA cm^−2^), the voltage for Cu_x_O@CuNiFeMoP/3D‐S remains as low as 2.07 V, substantially below that required by the 3D‐S electrode. To highlight the energy‐saving advantage of HMFOR, we directly compared the polarization behavior of the Cu_x_O@CuNiFeMoP/3D‐S//Pt/C system in HMFOR vs. conventional water electrolysis (Figure 5d). While the overall water splitting requires 2.50 V to reach 1000 mA cm^−2^, the HMFOR‐assisted process achieves the same current density at only 2.07 V, a remarkable reduction of 430 mV. We further evaluated the catalyst stability under industrial‐level current density, as shown in Figure S20. The Cu_x_O@CuNiFeMoP/3D‐S catalyst exhibits good stability, as evidenced by a negligible activity decay of approximately 0.1 mA cm^−2^ h^−1^ (a total decrease of 10 mA cm^−2^) over nine cycles exceeding 100 h of continuous testing. This pronounced voltage reduction underscores the exceptional energy efficiency offered by integrating OEORs with hydrogen production, particularly promising for industrial applications involving high‐concentration organic feedstocks (100 mm), establishing a practical pathway toward sustainable and cost‐effective electrochemical manufacturing.

**FIGURE 5:**
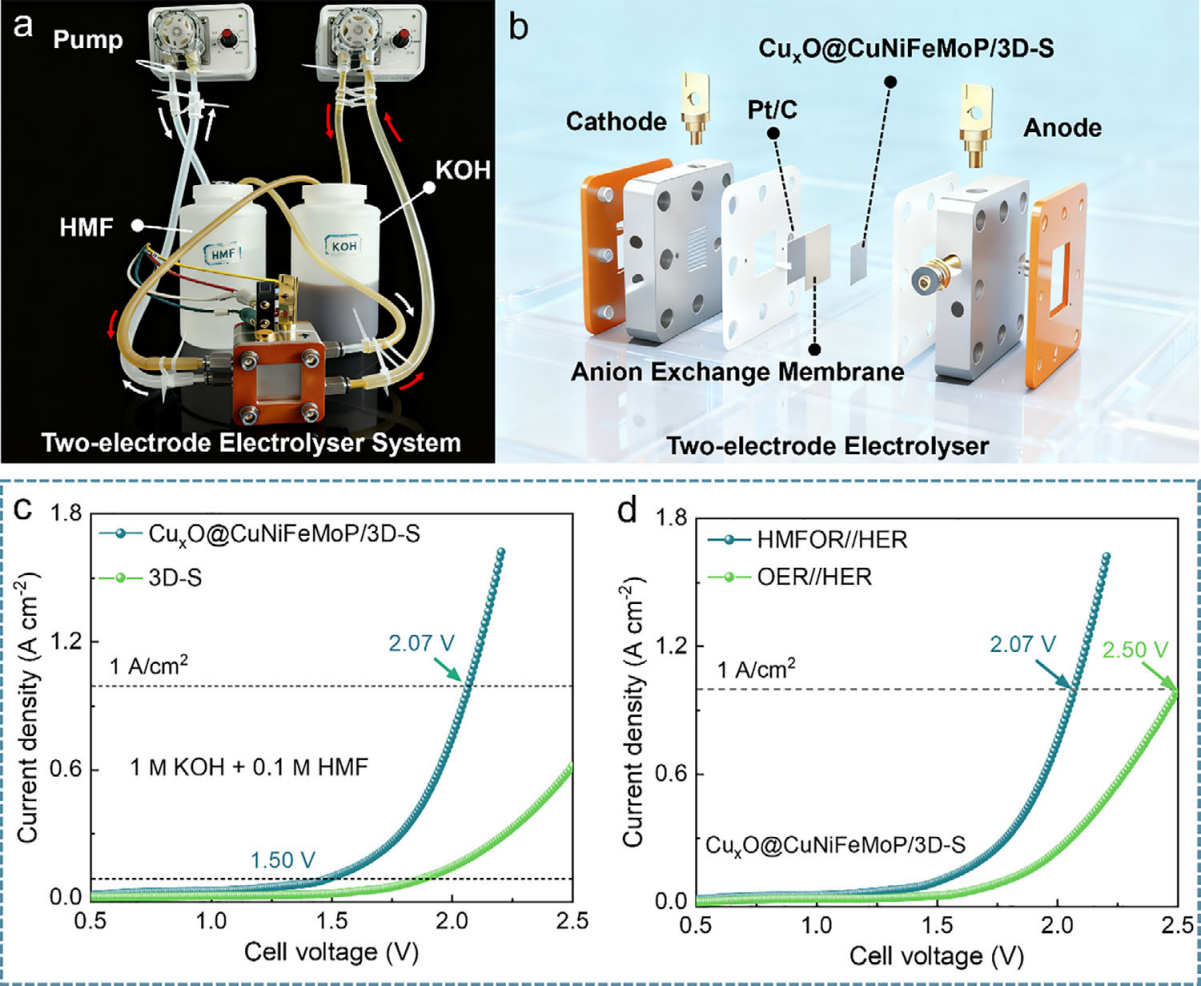
Two‐electrode HMFOR performance of Cu_x_O@CuNiFeMoP/3D‐S. (a) Schematic of the two‐electrode electrolyser system employing HMFOR as the anode reaction and HER as the cathode reaction. (b) Schematic of the two‐electrode electrolyser employing Cu_x_O@CuNiFeMoP/3D‐S as the anode catalyst and Pt/C as the cathode catalyst. (c) LSV curves of Cu_x_O@CuNiFeMoP/3D‐S and 3D‐S catalysts of the two‐electrode electrolyser. (d) LSV curves of Cu_x_O@CuNiFeMoP/3D‐S//Pt/C catalysts of the two‐electrode electrolyser for HMFOR and conventional water electrolysis.

Remarkably, the Cu_x_O@CuNiFeMoP/3D‐S catalyst demonstrates analogous synergistic enhancement in both GOR and UOR under high current density conditions, revealing a universal catalytic mechanism for diverse OEORs. The UOR and GOR act as parallel anodic reactions competing with OER during overall water electrolysis (Figure 6a). Similar to HMFOR, both UOR and GOR offer substantially lower thermodynamic potentials compared to water oxidation – 0.37 V for UOR (producing N_2_, CO_2_, and H_2_O, Figure 6b) [47] and 0.05 V for GOR (generating the valuable chemical gluconic acid, Figure 6c) [12] vs. 1.23 V for OER—providing energy‐efficient alternatives to conventional electrolysis [11, 48]. As shown in Figure 6d, the Cu_x_O@CuNiFeMoP/3D‐S catalyst demonstrates remarkable activity in GOR, particularly achieving a potential of only 1.44 V at high current density (500 mA cm^−2^)—representing a substantial 301 mV reduction compared to commercial IrO_2_ (1.75 V) (Figure 6e). Furthermore, we also observed identical phenomena in the UOR. The Cu_x_O@CuNiFeMoP/3D‐S electrode demonstrates remarkable UOR activity in 1 m KOH with 0.33 m urea, exhibiting a dramatically reduced overpotential compared to commercial IrO_2_ and the 3D‐S substrate. As evidenced by LSV measurements (Figure 6f), the catalyst achieves a 580 mV lower potential than IrO_2_ at 500 mA cm^−2^, highlighting its superior electrocatalytic performance. As shown in Figure 6g, Cu_x_O@CuNiFeMoP/3D‐S requires only 1.29 V to reach 10 mA cm^−2^, representing reductions of 140 and 170 mV vs. 3D‐S and IrO_2_, respectively. Notably, at industrial‐level current density (500 mA cm^−2^), it maintains an exceptionally low potential of 1.34 V, substantially lower than 3D‐S (1.63 V) and IrO_2_ (1.62 V). Kinetic analysis through Tafel plots (Figure 6h) demonstrates that Cu_x_O@CuNiFeMoP/3D‐S achieves a remarkably low slope of 20.99 mV dec^−1^, compared to 95.20 mV dec^−1^ for 3D‐S and 110.84 mV dec^−1^ for IrO_2_, indicating superior charge transfer kinetics. In addition, the *R*_ct_ from the EIS results of Cu_x_O@CuNiFeMoP/3D‐S is obviously smaller than that of the above‐mentioned other synthesized samples (Figure 6i), further facilitating faster electron transport at the electrode–electrolyte interface. As shown in Figure S21, the Cu_x_O@CuNiFeMoP/3D‐S electrode exhibits the highest double‐layer capacitance (*C*_dl_) value of 21.2 mF cm^−2—^approximately five times greater than that of the bare 3D‐S substrate (4.1 mF cm^−2^). This pronounced enhancement in *C*_dl_ directly correlates with a substantial increase in electrochemically active surface area (ECSA), demonstrating that the pulse‐electrodeposited Cu_x_O@CuNiFeMoP coral branch effectively creates a highly accessible interface for electrochemical processes. Furthermore, the Cu_x_O@CuNiFeMoP/3D‐S catalyst achieves a record‐low operational voltage of 1.34 V at 500 mA cm^−2^ among state‐of‐the‐art UOR electrocatalysts, as shown in Figure 6j. Critically, we further evaluated the catalyst stability under industrial‐level current density, as shown in Figure S22. The Cu_x_O@CuNiFeMoP/3D‐S electrode maintained stable operation at 500 mA cm^−2^ throughout the 280‐h durability test, demonstrating exceptional long‐term stability under high‐current‐density conditions. We have characterized the surface morphology and atomic structure of the catalyst after the long‐term stability test by SEM and TEM. The SEM images reveal that the coral‐like morphology of the CuxO@CuNiFeMoP/3D‐S catalyst is well‐preserved, indicating that no obvious exfoliation occurs on the catalyst surface after prolonged electrolysis (Figure S23). Furthermore, as shown in Figure S24, the Cu_x_O@CuNiFeMoP/3D‐S catalyst still retains the Cu_2+1_O phase and amorphous structure after the durability test. These results demonstrate that the heterostructure possesses outstanding structural stability under long‐term electrolysis conditions. Surprisingly, we find that the in situ Raman spectroscopy uncovers a voltage‐dependent evolution of Cu oxidation states that closely mirrors behavior observed during HMFOR (Figure 6k). At lower potentials, the coexistence of Cu^2+^ and Cu^+^ facilitates rapid redox cycling, whereas under high‐current conditions, Cu^+^ full oxidation to Cu^2+^ cooperatively activates Ni^3+^ sites. This dynamically coupled dual‐metal synergy establishes a universal catalytic paradigm for OEOR, underscoring the Cu_x_O@CuNiFeMoP/3D‐S exceptional versatility and consistent mechanistic behavior across diverse organic.

**FIGURE 6:**
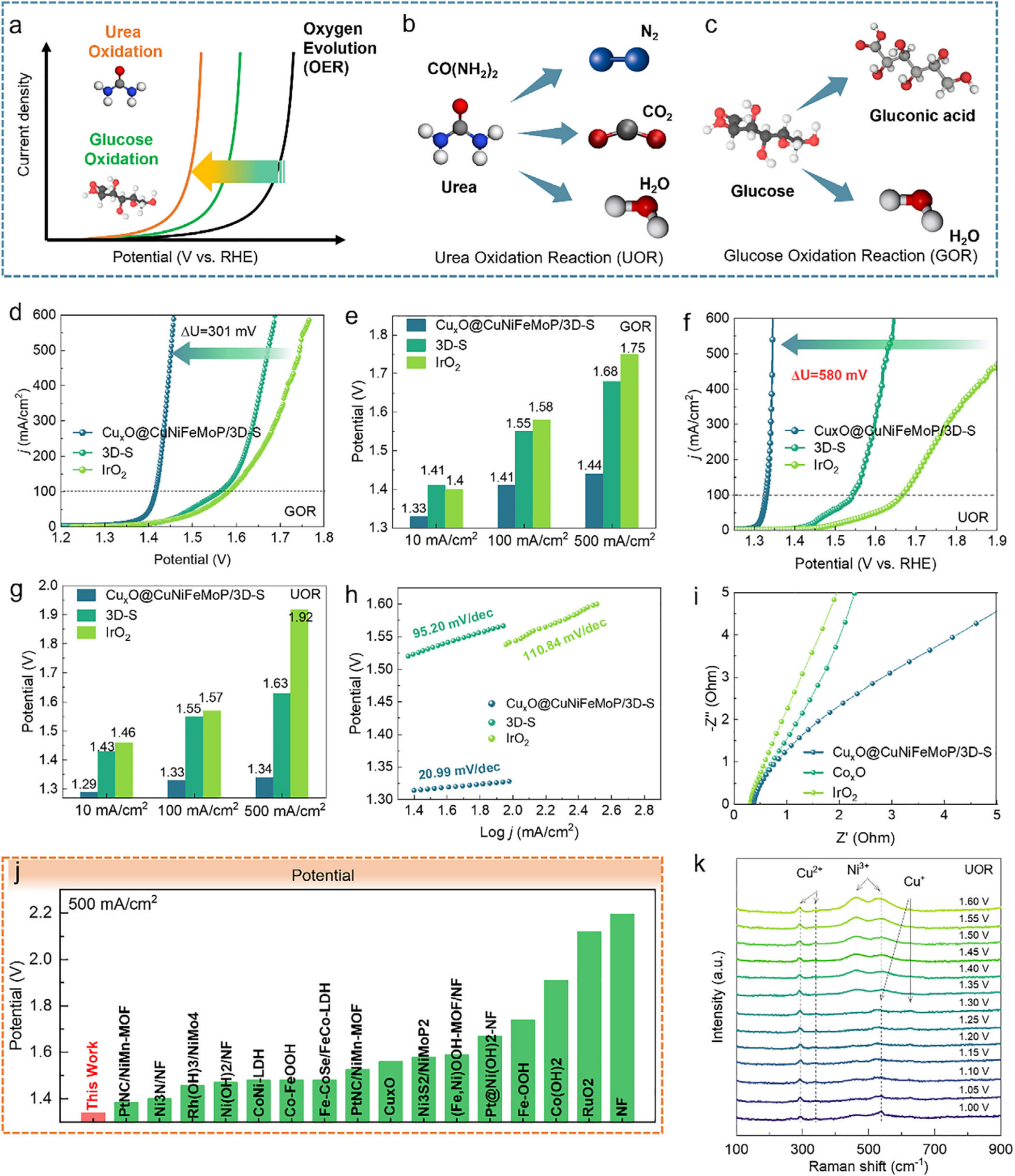
Diverse OEORs performance of the hierarchical Cu_x_O@CuNiFeMoP/3D‐S catalyst. (a–c) Schematic diagram of the diverse OEORs pathways (GOR and UOR). (d) LSV curves of Cu_x_O@CuNiFeMoP/3D‐S, 3D‐S, and IrO_2_ catalyst for GOR. (e) Histogram of potentials at 10, 100, and 500 mA cm^−2^ of different catalysts for GOR. (f) LSV curves of Cu_x_O@CuNiFeMoP/3D‐S, 3D‐S, and IrO_2_ catalyst for UOR. (g) Histogram of potentials at 10, 100, and 500 mA cm^−2^ of different catalysts for UOR. (h) Tafel curves of Cu_x_O@CuNiFeMoP/3D‐S, 3D‐S, and IrO_2_ catalysts for UOR. (i) EIS Nyquist plots of Cu_x_O@CuNiFeMoP/3D‐S, 3D‐S, and IrO_2_ catalyst for UOR. (j) Comparison of potential at 500 mA cm^−2^ of Cu_x_O@CuNiFeMoP/3D‐S catalyst for UOR. (k) SERS spectra obtained on Cu_x_O@CuNiFeMoP/3D‐S catalyst at different applied potentials for UOR.

## 3 Conclusions

In summary, this work demonstrates a comprehensive solution to the key challenge of high current density for universal OEORs through the development of a bio‐inspired hierarchical catalyst. First, we have established a coral‐inspired multiscale design strategy that integrates 3D‐printed TPMS scaffolds with PED active branches, creating a multi‐level composite catalyst that synchronously optimizes mass transport, active site density, mechanical stability, and hydrophilicity. Second, the catalyst achieves breakthrough performance in HMFOR, requiring only 1.47 and 1.57 V at the industrial‐grade current densities of 500 and 1000 mA cm^−2^, effectively resolving the high‐energy consumption challenges in OEORs at high current densities and significantly reducing the energy input for hydrogen production. Third, we discovered a novel synergistic catalytic mechanism, in which the coexistence of Cu^2^^+^ and Cu^+^ species together with Ni‐atom synergistic effects within the heterostructure enables sustained high activity under high‐current‐density conditions. Fourth, this mechanism demonstrates remarkable universality in various OEORs, as evidenced by exceptional GOR and UOR performance, especially achieving 500 mA cm^−2^ at just 1.34 V in UOR—thereby overcoming the limitation of conventional catalysts that are typically restricted to efficient operation under singular reaction conditions. Collectively, this study establishes a transformative strategy for developing high‐current OEOR catalysts, providing both a generalizable synthesis pathway and a universal mechanistic framework that significantly advances the practical implementation of energy‐saving hydrogen production.

## Author Contributions

**Chaoqun Pei**: methodology, investigation, formal analysis, writing – original draft, writing – review and editing. **Tao Zhang**: investigation, formal analysis, writing – review and editing, methodology. **Congrui Yang**: methodology, investigation, writing – review and editing. **Yutao Tan**: investigation, writing – review and editing, funding acquisition. **Zhengjie Chen**: methodology, investigation, validation, writing – review and editing. **Yuyang Qian**: investigation, methodology. **Xiaoyu Wu**: methodology, investigation. **Xiaodi Liu**: methodology, investigation. **Xiong Liang**: methodology, investigation. **Jie Dong**: methodology, investigation, funding acquisition. **Yongbin Guo**: investigation, validation. **Haibo Ke**: methodology, investigation, funding acquisition. **Wenqing Ruan**: methodology, investigation. **Zhenxuan Zhang**: methodology, investigation. **Tao Feng**: methodology, investigation, funding acquisition, writing – review and editing. **Jing Peng**: methodology, investigation, validation, writing – review and editing, funding acquisition. **Jiang Ma**: methodology, funding acquisition, writing – review and editing, supervision. **Baoan Sun**: conceptualization, methodology, investigation, validation, supervision, funding acquisition, writing – original draft, writing – review and editing, resources.

## Conflicts of Interest

The authors declare no conflicts of interest.

## Supporting information




**Supporting File**: advs78182‐sup‐0001‐SuppMat.docx.

## Data Availability

The data that support the findings of this study are available from the corresponding author upon reasonable request.
