# Supplementary material for: Coral‐Biomimetic Structured Organic Electro‐Oxidations With Exceptional High‐Current Catalytic Performance
Source: Adv Sci (Weinh). 2026 Oct 8:e78182. Online ahead of print. doi: 10.1002/advs.78182 (PMC13650022; doi:10.1002/advs.78182)
Supplement: Supplementary file 1 [file ADVS-9999-e78182-s001.docx]

**Supporting information for**

**Coral-Biomimetic Structured Organic Electro-oxidations With Exceptional**

**High-Current Catalytic Performance**

**Chaoqun Pei ^†^, Tao Zhang ^†^, Congrui Yang ^†^, Yutao Tan, Zheng-Jie Chen, Yuyang Qian, Xiaoyu Wu, Xiaodi Liu, Xiong Liang, Jie Dong, Yongbin Guo, Haibo Ke, Wenqing Ruan, Zhenxuan Zhang, Tao Feng , Jing Peng, Jiang Ma, Baoan Sun^*^**

^†^ These authors contributed equally to this work

* Corresponding Author:

Baoan Sun, email: sunba@iphy.ac.cn

**Table of Contents:**

**Methods**

**Fig.S1-S24**

**Supplementary Figures**

**Fig. S1 Morphological characterization of coral structure by SEM.**

**Fig. S2 The structural model of TPMS of 3D-S.**

**Fig. S3 Schematic diagram of the preparation of Cu_x_O@CuNiFeMoP/3D-S and the corresponded morphological characteristics by SEM.**

**Fig. S4 The mass transfer simulation results of the TPMS structure.**

**Fig. S5 Spatial variation of local porosity and directional flow velocities within the Gyroid architecture.**

**Fig. S6 The simulation results of the mechanical properties of the TPMS structure.**

**Fig. S7 The hydrophilicity and aerophobicity properties of the coral-like Cu_x_O@CuNiFeMoP/3D-S catalyst.**

**Fig. S8 XRD results of CuxO@CuNiFeMoP/3D-S and 3D-S catalysts.**

**Fig. S9 Cu K-edge XANES spectra of Cu foil, Cu_2_O, CuO, and Cu_x_O@CuNiFeMoP/3D-S.**

**Fig. S10 LSV curves in 1 M KOH with 50 mM HMF of Cu_x_O@CuNiFeMoP/3D-S and Ni(OH)_2_.**

**Fig. S11 LSV curves in 1 M KOH with 50 mM HMF of Cu_x_O@CuNiFeMoP/3D-S and Cu_x_O@CuNiFeMoP/NF.**

**Fig. S12 LSV curves in 1 M KOH with 50 mM HMF of varied compositions, CuNiFeMoP, CuNiMoP and CuNiP.**

**Fig. S13 The specific activity (mA cm^-2^₍_ECSA_₎) of Cu_x_O@CuNiFeMoP/3D-S and 3D-S catalysts.**

**Fig. S14 Time-resolved chromatograms tracked the temporal evolution of these species throughout electrolysis of different potential.**

**Fig. S15 Calibration curves for high-performance liquid chromatography were first constructed with analytical-grade HMF and its electro-oxidation congeners.**

**Fig. S16 Statistical data on the selectivity of different catalysts in the organic electro-oxidation.**

**Fig. S17 Faradaic efficiency of FDCA over the full current‑density range.**

**Fig. S18 Adsorption energies of Cu_x_O@CuNiFeMoP/3D-S for HMF and OH^-^.**

**Fig. S19 Catalytic enhancement mechanism.**

**Fig. S20 Long-term stability testing results for HMFOR in AEM devices for over 100 h.**

**Fig. S21 *C*_dl_ results of Cu_x_O@CuNiFeMoP/3D-S and 3D-S catalysts.**

**Fig. S22 The stability test performance of the Cu_x_O@CuNiFeMoP/3D-S catalyst under 280 hours of operation.**

**Fig. S23 Characterized the surface morphology of the catalyst after long‑term stability test by SEM.**

**Fig. S24 Characterized the atomic structure of the catalyst after long‑term stability test by TEM.**

**Methods**

**Catalyst preparation.**

Stainless steel powders produced by gas atomization served as the starting material for fabricating 3D-S substrates through micro-laser powder bed fusion (μLPBF). The powder particles, characterized by diameters between 0 and 53 μm, demonstrated optimal morphology for LPBF processing, including spherical shape consistency, homogeneous size distribution, and smooth surface quality. Fabrication of metallic glass substrates was conducted using a BLT-S200GX μLPBF apparatus, which features a precision laser focus diameter of 30 μm—significantly finer than conventional LPBF systems—enabling manufacturing of high-definition structures. The setup incorporates a 500 W IPG fiber laser source and maintains an argon-filled processing environment with oxygen content rigorously controlled below 100 ppm. Critical manufacturing parameters were established at 80 W laser power, 1200 mm/s scan speed, and 20 μm layer thickness. The Cu_x_O@CuNiFeMoP (Cu_55_Ni_20_Fe_10_Mo_10_P_5_) catalyst was subsequently deposited onto the 3D-S working electrode via PED in a standard three-electrode configuration. The deposition was carried out at a pulsed current density of 150 mA cm^-2^ for 30 minutes. The electrolyte solution was prepared by dissolving the following reagents in 200 mL of deionized water: 4.43 g NiSO_4_∙6H_2_O, 2.0 g Na_2_MoO_4_∙2H_2_O, 1.5 g FeSO_4_∙7H_2_O, 1.79 g NaH_2_PO_4_, 0.5 g CuSO_4_∙5H_2_O, 2.63 g NaCl, 0.07 g sodium dodecyl sulfate (SDS), and 5.3 g trisodium citrate. Prior to deposition, the solution was continuously purged with nitrogen gas under constant stirring to ensure homogeneity and prevent oxidation of metal precursors.

**Characterizations.**

The structural and compositional characteristics of the microwires were systematically characterized using multiple analytical techniques. Phase identification was performed by *X*-ray diffraction (XRD, BRUKER D8 ADVANCE/DISCOVER) with Cu-*K*_α_ radiation (*λ* = 1.54 Å). Morphological features were examined using scanning electron microscopy (SEM, IT500). Microstructural analysis was conducted by transmission electron microscopy (TEM, FEI Tecnai G2 F30 S-Twin) operated at 300 kV, coupled with energy-dispersive X-ray spectroscopy (EDX) for elemental mapping. Surface wetting behavior was evaluated through contact angle measurements using a surface tension analyzer (Dataphysics OCA20). For in situ spectroscopic analysis, Raman measurements were acquired on a Horiba LabRAM HR Evolution system with a 532 nm laser (600 gr/mm grating, 1298 nm spot size), where applied potentials were incrementally increased from 1.00 V to 1.60 V in 0.05 V steps with 2-minute stabilization at each potential. Complementary in situ *X*-ray photoelectron spectroscopy (XPS) was performed on a Thermo Fisher ESCALAB XI+ instrument equipped with a monochromatic Al Kα source (300 W) and Phoibos150 MCD-9 detector at 35° take-off angle under potential-controlled conditions.

**Electrochemical measurement.**

A CHI 660E electrochemical workstation (CH Insrument) is used for electrochemical testing with a three-electrode system (prepared electrodes as working electrodes, Pt plate as the counter electrode, and Hg/HgO as the reference electrode). **For HMFOR**, High-performance liquid chromatography (HPLC) was employed to quantify the HMFOR products in 1.0 M KOH with 10 mM HMF. To quantitatively determine the intermediates, final products, and associated Faradaic efficiencies (FE) during the electrochemical oxidation of HMF, CuxO@CuNiFeMoP/3D-catalyzed reactions were conducted in 25 mL of 1.0 M KOH containing 10 mM HMF at 1.45 V vs. RHE. The theoretical charge for full HMF-to-FDCA conversion is ~144.7 C, near-quantitative HMF oxidation efficiency under the prevailing kinetic competition . OER polarization curves verified negligible oxygen evolution at 1.45 V, confirming selective HMF oxidation under these conditions. Calibration curves for HPLC were first constructed with analytical-grade HMF and its electro-oxidation congeners. (Fig. S15). Time-resolved chromatograms tracked the temporal evolution of these species throughout electrolysis (Fig. S14-S15). The UOR measurements were conducted in 1 M KOH electrolyte supplemented with 0.33 M urea. Prior to testing, the electrolyte was saturated with high-purity oxygen to establish and maintain the O₂/H₂O equilibrium at 1.23 V vs. RHE throughout the experiment. All catalysts underwent electrochemical preconditioning via 20 cyclic voltammetry cycles at a scan rate of 100 mV s^-1^. Linear sweep voltammetry was subsequently performed from 0.2 to 0.8 V vs. Hg/HgO at 5 mV s^-1^ to evaluate UOR activity. All reported potentials were converted to the reversible hydrogen electrode (RHE) scale using the Nernst equation (*E*_RHE_ = *E*_SCE_ + 0.059 × pH + 0.098) (50% iR‑compensation). Electrochemical impedance spectroscopy was measured at open-circuit potential over a frequency range of 100 kHz to 0.01 Hz. Catalyst stability was assessed through chronoamperometry at 1.34 V vs. RHE for 280 hours, maintaining a current density of 500 mA cm^-2^.

**Computational Methods**

Spin-polarized density functional theory calculations were performed using the Vienna Ab initio Simulation Package (VASP). The projector-augmented wave method and the Perdew-Burke-Ernzerhof exchange-correlation functional were employed. Dispersion interactions were described using the DFT-D3 correction. The plane-wave cutoff energy was set to 450 eV. The convergence criteria for the electronic energy and atomic forces were 1.0 x 10^-5^ eV and 0.02 eV A^-1^, respectively. A 3 x 3 x 1 k-point mesh was used for the crystalline oxide surface, whereas Gamma-point sampling was adopted for the large amorphous and heterostructure models.

A vacuum layer of at least 15 A was introduced perpendicular to the surface. A 100-atom Cu_55_Ni_20_Fe_10_Mo_10_P_5_ amorphous model was generated using a melt-quench procedure and subsequently optimized by DFT. The heterostructure was constructed by contacting the defective Cu_2+1_O (200) slab with the relaxed amorphous alloy. Several initial interfacial separations and lateral configurations were tested, and the most stable relaxed interface was selected.

**Fig.S1 Morphological characterization of coral structure by SEM.**

**Fig.S2 The structural model of TPMS of 3D-S.**

**Fig. S3 Schematic diagram of the preparation of Cu_x_O@CuNiFeMoP/3D-S and the corresponded morphological characteristics by SEM.** **a,** Schematic diagram of 3D-S preparation through 3D printing and the morphological images. **b,** Schematic diagram of Cu_x_O@CuNiFeMoP preparation through pulsed electrodeposition method and the morphological images.

**Fig. S4 The mass transfer simulation results of the TPMS structure.** **a,** Schematic illustration of the fluid flow simulation, showing flow direction through the 3D-reconstructed lattice model. **b,** XY-dimensional velocity contour maps of a representative cross-section, showing localized flow acceleration zones (red) and stagnation regions (blue). **c,** XZ-dimensional velocity contour maps of a representative cross-section

**Fig. S5 Spatial variation of local porosity and directional flow velocities within the Gyroid architecture.** a, Local porosity variation along the selected transport path. b–d, Corresponding mean velocity components along the x, y, and z directions, respectively.

**Fig.S6 The simulation results of the mechanical properties of the TPMS structure.**

**Fig. S7 The hydrophilicity and aerophobicity properties of the coral-like Cu_x_O@CuNiFeMoP/3D-S catalyst. a-b,** The hydrophilicity properties of the coral-like Cu_x_O@CuNiFeMoP/3D-S and 3D-S catalyst. **c-d,** The aerophobicity properties of the coral-like Cu_x_O@CuNiFeMoP/3D-S and 3D-S catalyst.

**Fig. S8 XRD results of CuxO@CuNiFeMoP/3D-S and 3D-S catalysts.**

**Fig. S9 Cu K-edge XANES spectra of Cu foil, Cu_2_O, CuO, and Cu_x_O@CuNiFeMoP/3D-S.**

**Fig. S10 LSV curves in 1 M KOH with 50 mM HMF of Cu_x_O@CuNiFeMoP/3D-S and Ni(OH)_2_.**

**Fig. S11** **LSV curves in 1 M KOH with 50 mM HMF of Cu_x_O@CuNiFeMoP/3D-S and Cu_x_O@CuNiFeMoP/NF.**

**Fig. S12 LSV curves in 1 M KOH with 50 mM HMF of varied compositions, CuNiFeMoP, CuNiMoP and CuNiP.** a, CuNiP and CuNiMoP. b, LSV curves in 1 M KOH with 50 mM HMF of varied compositions.

**Fig. S13 The specific activity (mA cm^-2^₍_ECSA_₎) of** **Cu_x_O@CuNiFeMoP/3D-S and 3D-S catalysts.**

**Fig. S14 Time-resolved chromatograms tracked the temporal evolution of these species throughout electrolysis of different potential. a,** 1.42 V. **b,** 1.47 V.

**Fig. S15 Calibration curves for high-performance liquid chromatography were first constructed with analytical-grade HMF and its electro-oxidation congeners. a,** HMF. **b,** DFF. **c,** HMFCA. **d,** FFCA. **e,** FDCA.

**Fig. S16 Statistical data on the selectivity of different catalysts in the organic electro-oxidation.**

**Fig. S17** **Faradaic efficiency of FDCA over the full current‑density range.** a, The concentrations of HMF and its various products. b, Faradaic efficiency of FDCA over the full current‑density range.

**Fig. S18 Adsorption energies of Cu_x_O@****CuNiFeMoP/3D-S for HMF and OH^-^.**

**Fig. S19 Catalytic enhancement mechanism.** a, PDOS of Ni 3d of Cu_x_O@CuNiFeMoP and CuNiFeMoP. b, Local difference charge integration of different samples.

**Fig. S20 Long-term stability testing results for HMFOR in AEM devices for over 100 h.**

**Fig. S21 *C*_dl_ results of Cu_x_O@CuNiFeMoP/3D-S and 3D-S catalysts.**

**Fig. S22 The stability test performance of the Cu_x_O@CuNiFeMoP/3D-S catalyst under 280 hours of operation**

**Fig. S23** **Characterized the surface morphology of the catalyst after long‑term stability test by SEM.**

**Fig. S24 Characterized the atomic structure of the catalyst after long‑term stability test by TEM.**
